# Isokinetic Characteristics of Amateur Boxer Athletes

**DOI:** 10.3389/fphys.2018.01597

**Published:** 2018-11-14

**Authors:** Ioannis Tasiopoulos, Pantelis T. Nikolaidis, Alexandra Tripolitsioti, Apostolos Stergioulas, Thomas Rosemann, Beat Knechtle

**Affiliations:** ^1^Faculty of Human Movement and Quality of Life, University of Peloponnese, Sparta, Greece; ^2^Exercise Physiology Laboratory, Nikaia, Greece; ^3^Institute of Primary Care, University of Zurich, Zürich, Switzerland; ^4^Medbase St. Gallen Am Vadianplatz, St. Gallen, Switzerland

**Keywords:** strength, shoulders rotators, isokinetic, eccentric, concentric

## Abstract

**Aim:** The kinetic chain of the punch of boxers is characterized by the contribution of the shoulder; however, the isokinetic muscle strength of shoulder’s rotators muscles has not been well studied so far, especially with regards to performance. Therefore, the aim of the present study was (a) to profile the isokinetic muscle strength of rotators of the glenohumeral joint, bilateral (BL), unilateral (UL) and functional ratios in amateur boxers, and (b) to examine the variation of these muscle strength characteristics by performance level.

**Methods:** Forty male amateur Greek boxers from three division levels (elite, *n* = 22; second division, *n* = 11; and third division, *n* = 7), and 10 non-athletes (control group) were tested, using the isokinetic dynamometer Kin-Com^TM^ in the scapular seated position under standard conditions. We examined (i) peak torque (PT) of internal (IR) and external (ER) rotators during concentric (CON) and eccentric (ECC) contractions at low (60°/s), medium (120°/s), and high speed (180°/s), (ii) BL, (iii) UL, and (iv) functional ratios of dominant (D) and non-dominant (ND) limbs.

**Results:** Boxers were stronger than control group in IR and ER at all speeds, and D outscored ND limb (*p* < 0.05). Elite boxers were stronger than group B and C (*p* < 0.05); however, when peak torque was expressed in relative to body mass values, these differences were attenuated. The BL ratios for the men athletes were under 10% at 60 and 180°/s, the UL ratios were lower at 60 and higher at 180°/s and significant lower in the dominant limb at all speeds. The functional ratios of IR ECC / ER CON were higher between the control group at all speeds and the ER ECC/IR CON ratios were between 60 and 180°/s, and the two limbs with higher values at 180°/s.

**Conclusion:** Boxers had the strongest dominant limb and their BL ratios were normal at all speeds except of 120°/s ECC. The UL ratios of ECC at 120 and 180°/s of ND were normal, and at the other speeds abnormal due to high IR. In addition, the functional ratios may be related to the kinetic chain of the punch, which in turn correlates with the contribution of ER of both limbs.

## Introduction

In boxing the impact of strength has an important role in the physiological profile (Chaabène et al., 2014), because the win of the round depends on the delivery of clearly and powerful punches to the opponent’s target areas. Biomechanics and kinematics analysis of the fist is characterized by the combination of the ankle, thigh, trunk, and the forearm (Stojsih et al., 2010), with similar kinetic chain as that of the throwing arm of baseball athletes (Fleisig et al., 1996). Few researchers have assessed the isometric and isokinetic muscle strength of boxers’ legs, trunks, shoulders, and elbows (Lenetsky et al., 2015). In their studies, they observed correlation between flexibility of the trunk and strength of the punches, asymmetry on the strength of the legs, symptoms that increase the risk of injury of the upper limb.

Muscles imbalances of IR and ER of shoulder are important for the injury prevention, with the ratio of ER/IR for healthy subjects in low isokinetic concentric velocity at 30–60°/s being 2/3 (Heyward and Gibson, 2014). The differences at high and very high speeds might depend on kinetic movement of the sports (Edouard et al., 2013). The rotator cuff muscles have a vital role in maintaining normal arthrokinematic and asymptomatic shoulder function. Their main role is joint stability by exerting negative pressure from the head of the bone in the upper shoulder, in order the two bones to apply tight between them, while the muscles stabilize and help preventing further instability (Speer, 1995). Moreover, the glenohumeral stability depends on the ratio of forces shifting in several directions and compression forces; thus, a change in the ratio between these forces alters the instability of the joint (Labriola et al., 2005). Accordingly, an optimal balance of strength between the external and the internal rotators is necessary for the normal function of the articulation of the shoulder, especially during sports activities (Ellenbecker and Mattalino, 1997).

The myodynamic bilateral ratios from 0 to 10% are considered normal, 10–20% possibly abnormal, whereas greater than 20% abnormal (Ellenbecker and Davies, 2000). When these ratios are greater than 15%, there is 2.6 times greater possibility of injury in the weakest limb (Knapik et al., 1991). However, this topic has not been examined in isokinetic muscle shoulder rotators at low to high speeds in boxing. Therefore, the aim of the present study was to examine the relationship of isokinetic strength of rotator muscles of glenohumeral joint and performance in amateur boxers (a) compared to non-athletes and (b) of different performance levels.

## Materials and Methods

Forty male amateur Greek boxers (age 25.5 ± 3.5 years; height 177 ± 6 cm; body weight 78.8 ± 8.8 kg) and 10 non-athletes (control group) participated voluntarily by competing in the National Hellenic Boxing Championship (Table 1). Boxers were divided in three performance groups depending on the level of their championship division: Elite (*n* = 22), B (second division, *n* = 11), C (third division, *n* = 7). All boxers had sport experience more than three years and the number of played games was more than 35 in Elite, 16–34 in B, and 0–15 in C group.

**Table 1:** Physical characteristics of subjects by performance group (Elite, B and C).

Group (*n*)	Age (yrs)	Height (cm)	Weight (kg)
Elite (22)	25.7 (2.9)	179 (6)	80.6 (8.3)
B (11)	26.4 (4.1)	176 (5)	75.4 (6.5)
C (7)	22.9 (3.6)	176 (8)	78.4 (12.5)
Total (40)	25.5 (3.5)	178 (6)	78.8 (8.8)
Control (10)	22.0 (2.0)	172 (6)	70.6 (5.5)

*Values are presented as means with standard deviations in brackets.*

All participants were free of injury for at least 6 months prior to testing procedures, having no previous experience in isokinetic testing of the shoulder. They were informed about testing procedures, the benefits and potential risks of research, and signed informed consent form in accordance with the guidelines on human rights prior to testing session. They were examined at the beginning of training year (September 2011–2015). The institutional review board of the Faculty of Human Movement & Quality of Life, Peloponnese University, Sparta, Greece, approved all procedures of this study.

The isokinetic dynamometer Kin-Com^TM^ (Chattem, Chattanooga, TN, United States) was used for testing and was calibrated at 60, 120, and 180°/s. Outcome measures were (i) peak torque (PT) of concentric (con) and eccentric (ecc) contractions of dominant (D) and non-dominant (ND) limbs, (ii) bilateral (BL), (iii) unilateral (UL), and (iv) functional ratios. The evaluation of the shoulder using isokinetic dynamometry has demonstrated high to very high reliability, 0.74–0.97 (Edouard et al., 2011).

Each participant performed a 5-min standard warm up (Ellenbecker and Mattalino, 1997). Thereafter, the isokinetic tests were performed on seated position with 45° of shoulder abduction in the scapular plane which was considered as the most reliable for IR and ER strength assessment and provided more anatomical advantages (Edouard et al., 2011). The trunk and the hip were stabilized through two straps that were cross-placed in front of the chest and fixed to the back of the chair. The hip was stabilized through a strap about 45° throughout the pelvis and fixed on the chair to avoid additional movement, with the seat to mobile stabilized where found the best location test. The limb measurements (i.e., first left or right) was in a random order and the elbow placed at 90° flexion, supported by using strap. The length of the lever arm to anatomically was defined for each participant, and the weight of each limb was measured and used for gravity correction (Edouard et al., 2009).

**FIGURE 1:**
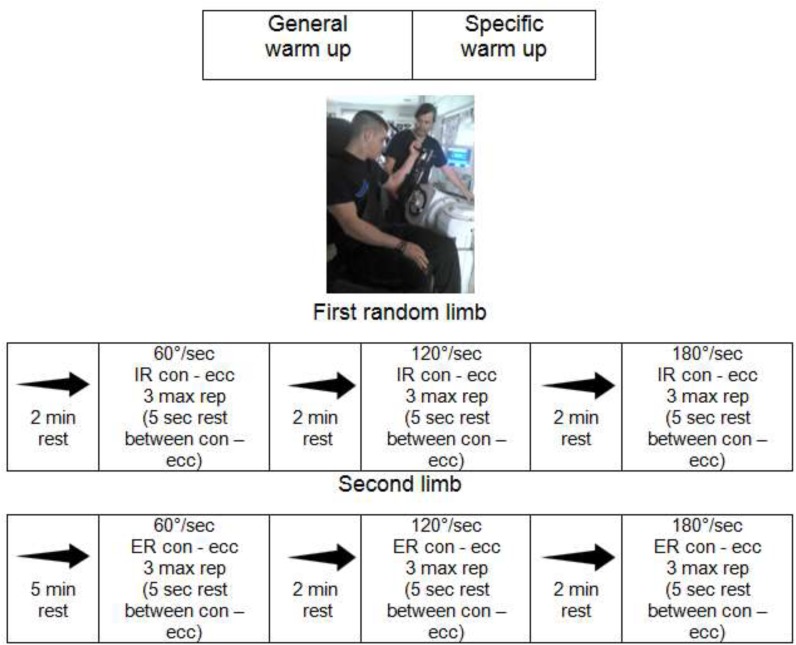
Experimental set-up. max, maximum; rep, repetitions; IR, internal rotator; ER, external rotator; 60, 120, and 180°/s, angular velocity; con, concentric; ecc, eccentric.

Before considering the maximal PT, participants performed a specific warm-up consisting of five repetitions (including three submaximal and two maximum con/ecc contractions) according to a recommended intermittent warm-up protocol (Keskula and Perrin, 1994). The evaluation process consisted of three repetitions of the maximal voluntary con and ecc contraction of IR and ER in angle of 60, 120, and 180°/s. Every con maximum contraction was measured first, followed by the measurement of an ecc. There were two minutes of rest between tests of two muscle groups and each subject was tested first at 60°, 120°, and 180°/s (Wilhite et al., 1992), with two minutes rest between the angle, five second rest between con and ecc and five minutes rest between the two shoulders (Figure 1).

The results of PT of con and ecc were expressed in both absolute (N.m) and relative to body mass values (N.m.kg^-1^), with the range of motion for the IR from 90 to 0° degrees and vice versa for ER 0 to 90°. The minimum strength value was set at 25 N for all measurements. Furthermore, BL (%) was calculated using the formula 100 × [PT(D)–PT(ND)]/PT(D), whereas UL (%) was 100 × ER/IR. Two functional ratios were considered: (a) IRecc/ERcon, and (b) ERecc/IRcon.

### Statistical Analysis

The statistical analyses were conducted using the statistical package SPSS v IBM 23.0 (SPSS, Chicago, IL, United States 223). The average of the three attempts in each test was used for further analysis. For the normality of the data we used the Kolmogorov-Smirnov test. The data were expressed as means and standard deviations (SD). The analysis of the measurements was performed using descriptive statistics, and one-way analysis of variance (ANOVA) and *post hoc* Bonferroni correction. A repeated measures ANOVA compared boxers and control group for all tests. The magnitude of these differences was examined using effect size η^2^ and was evaluated as small (0.010 < η^2^ ≤ 0.059), moderate (0.059 < η^2^ ≤ 0.138) and large (η^2^ > 0.138). In addition, a dependent *t*-test examined differences between dominant and non-dominant limbs. Significance was set at alpha = 0.05.

## Results

### Peak Torque

#### Boxers Versus Control Group

**FIGURE 2:**
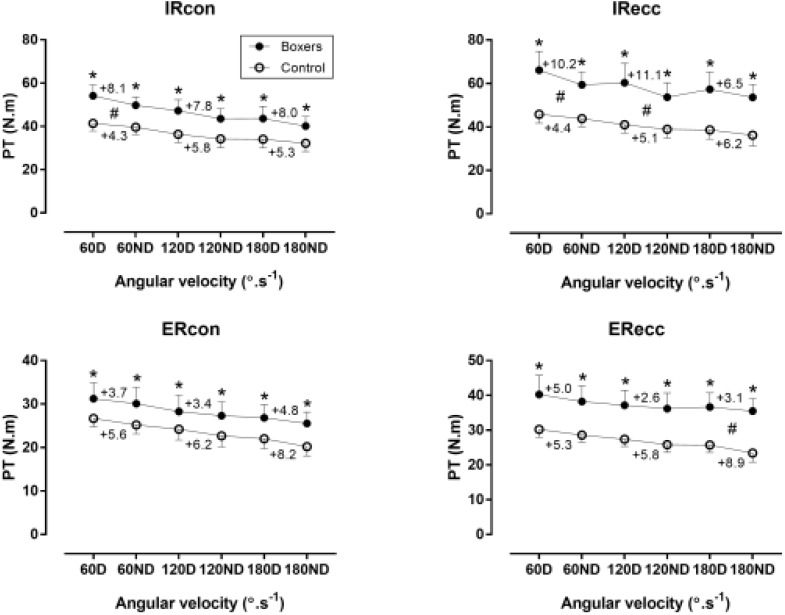
Peak torque (PT) of internal (IR), and external rotators (ER) of shoulder against 60, 120, and 180°/s in boxers and control group. ^∗^difference between boxers and control group at *p* < 0.05. D, dominant limb; ND, non-dominant limb. The numbers between D and ND points denote percentage differences between D and ND values. #limb × group interaction on peak torque at *p* < 0.05.

A large main effect of sport on PT was observed (*p* < 0.01), with boxers outscoring control group for IR at all velocities in both limbs (η^2^ = 0.34–0.60) (Figure 2). In addition, a large main effect of sport on PT was shown in ER (*p* < 0.01), with boxers presenting higher score than control group at all velocities in both limbs (η^2^ = 0.18–0.70). Peak torque in relative to body mass values of boxers and control group can be seen in Table 2. Boxers outscored control group for all conditions except concentric action of external rotators.

**Table 2:** Peak torque (in N.m.kg^-1^) values in internal (IR) and external rotators (ER) of shoulders in boxers and control group.

	IR				ER			
	con		ecc		con		ecc	
	Boxers	Control	Boxers	Control	Boxers	Control	Boxers	Control
60D	0.69 ± 0.08^‡^	0.59 ± 0.04	0.85 ± 0.13^‡^	0.65 ± 0.04	0.40 ± 0.06	0.38 ± 0.03	0.52 ± 0.08^†^	0.43 ± 0.03
60ND	0.64 ± 0.08^†^	0.56 ± 0.04	0.76 ± 0.11^‡^	0.62 ± 0.04	0.38 ± 0.05	0.36 ± 0.03	0.49 ± 0.07^†^	0.41 ± 0.03
120D	0.60 ± 0.08^†^	0.51 ± 0.04	0.77 ± 0.12^‡^	0.58 ± 0.04	0.36 ± 0.06	0.34 ± 0.03	0.48 ± 0.07^‡^	0.39 ± 0.02
120ND	0.56 ± 0.07^†^	0.48 ± 0.04	0.69 ± 0.10^‡^	0.55 ± 0.04	0.35 ± 0.05	0.32 ± 0.03	0.46 ± 0.07^‡^	0.37 ± 0.03
180D	0.56 ± 0.08^†^	0.48 ± 0.05	0.73 ± 0.11^‡^	0.55 ± 0.05	0.34 ± 0.05	0.31 ± 0.03	0.47 ± 0.06^‡^	0.37 ± 0.03
180ND	0.51 ± 0.07^∗^	0.46 ± 0.05	0.69 ± 0.10^‡^	0.51 ± 0.06	0.33 ± 0.04^†^	0.29 ± 0.02	0.45 ± 0.05^‡^	0.33 ± 0.04

*Symbols denote differences (^∗^p < 0.05, ^†^p < 0.01, ^‡^p < 0.001) between groups.*

#### Boxers by Performance Level

A large main effect of performance group on PT was found (*p* < 0.01) with elite boxers showing the highest score in IRcon at 60D (*p* < 0.01, η^2^ = 0.34), 60ND (η^2^ = 0.32), 120D (η^2^ = 0.33), 180D (η^2^ = 0.35), IRecc at 60D (η^2^ = 0.27), 60ND (η^2^ = 0.26), 120D (η^2^ = 0.42), 120ND (η^2^ = 0.35), 180D (η^2^ = 0.59), and 180ND (η^2^ = 0.47) (Table 3). No difference among performance groups was observed in IRcon at 120ND (*p* > 0.05, η^2^ = 0.14) and 180ND (*p* > 0.05, η^2^ = 0.09).

**Table 3:** Peak torque (in N.m) values in internal (IR) and external rotators (ER) of shoulders among groups of athletes (Elite, B and C).

	IR						ER					
	con			Ecc			con			ecc		
	Elite	B	C	Elite	B	C	Elite	B	C	Elite	B	C
60D	56.7 ± 4.8^∗†###^	51.7 ± 3.7^##^	49.9 ± 2.0	68.5 ± 7.5^†###^	67.6 ± 8.9^†##^	56.4 ± 5.0^#^	32.1 ± 2.9	31.2 ± 4.4^#^	28.6 ± 3.7	42.0 ± 4.2†^##^	40.7 ± 5.4†^#^	34.1 ± 5.7
60ND	51.7 ± 4.0^∗^†	47.4 ± 1.8	47.3 ± 2.3	61.2 ± 5.5†	59.9 ± 5.6†	52.9 ± 3.7	31.1 ± 3.7	29.9 ± 3.4	27.3 ± 3.6	40.1 ± 3.1†	38.2 ± 3.7†	32.4 ± 4.5
120D	49.8 ± 5.3^∗^†^###^	44.8 ± 2.8^##^	43.0 ± 1.2	64.6 ± 7.5†^###^	59.0 ± 7.3†^###^	49.1 ± 2.9	29.5 ± 3.6†^#^	28.4 ± 3.2†^#^	24.3 ± 2.8	39.3 ± 3.6†	36.5 ± 2.8†	31.4 ± 2.1
120ND	45.1 ± 5.3	41.5 ± 3.0	42.0 ± 3.2	56.5 ± 5.8†	53.1 ± 5.6†	45.9 ± 4.0	28.5 ± 2.9†	26.9 ± 2.6	24.3 ± 3.0	38.6 ± 4.1^∗^†	35.1 ± 1.9†	30.3 ± 1.6
180D	46.6 ± 5.0^∗^†^###^	40.6 ± 3.6^#^	38.9 ± 5.2	62.0 ± 5.8^∗^†^###^	54.9 ± 4.6†^##^	46.1 ± 3.5	28.2 ± 2.8^∗^†^###^	25.3 ± 2.2^##^	25.0 ± 2.8	39.5 ± 2.8^∗^†^###^	34.8 ± 1.5†^##^	30.7 ± 1.1
180ND	41.1 ± 4.0	38.0 ± 3.5	40.0 ± 6.2	56.9 ± 4.5^∗^†	51.6 ± 5.3	46.3 ± 2.4	26.6 ± 2.4^∗^	23.7 ± 1.9	25.0 ± 2.8	38.0 ± 2.7^∗^†	33.5 ± 1.8	31.0 ± 1.2

*IR, internal rotation; ER, external; con, concentric; ecc, eccentric; D, dominant; ND, non-dominant; 60,120, and 180, angular velocity. ^∗^ and † difference from B and C, respectively, at p < 0.05. Differences between D and ND were presented as ^#^p < 0.05, ^##^p < 0.01, and ^###^p < 0.001).*

Furthermore, a large main effect of performance group on PT was shown (*p* < 0.05), with elite boxers outscoring group B and C in ERcon at 120D (η^2^ = 0.25), 120ND (η^2^ = 0.24), 180D (η^2^ = 0.26), and 180ND (η^2^ = 0.23), ERecc at 60D (η^2^ = 0.27), 60ND (η^2^ = 0.40), 120D (η^2^ = 0.46), 120ND (η^2^ = 0.48), 180D (η^2^ = 0.7) and 180ND (η^2^ = 0.60). No difference among performance groups was observed in ERcon at 60D (*p* > 0.05, η^2^ = 0.13) and 60ND (*p* > 0.05, η^2^ = 0.14). When peak torque was expressed in relative to body mass values, Elite and B group were stronger than group C in eccentric action of internal and external rotators; however, no difference in concentric action of both rotators was shown among groups (Table 4).

**Table 4:** Peak torque in relative to body mass values (N.m.kg^-1^) in internal (IR) and external rotators (ER) of shoulders by performance group (Elite, B and C).

	IR						ER					
	con			Ecc			con			ecc		
	Elite	B	C	Elite	B	C	Elite	B	C	Elite	B	C
60D	0.71 ± 0.08	0.69 ± 0.07	0.65 ± 0.12	0.85 ± 0.10	0.90 ± 0.14^C^	0.74 ± 0.13	0.40 ± 0.04	0.42 ± 0.08	0.37 ± 0.06	0.52 ± 0.05	0.55 ± 0.10^C^	0.45 ± 0.11
60ND	0.65 ± 0.08	0.63 ± 0.05	0.62 ± 0.12	0.76 ± 0.09	0.80 ± 0.11	0.69 ± 0.14	0.39 ± 0.04	0.40 ± 0.06	0.35 ± 0.06	0.50 ± 0.05^C^	0.51 ± 0.07^C^	0.42 ± 0.10
120D	0.62 ± 0.07	0.60 ± 0.06	0.56 ± 0.09	0.81 ± 0.10^C^	0.79 ± 0.11^C^	0.64 ± 0.10	0.37 ± 0.06	0.38 ± 0.05	0.31 ± 0.05	0.49 ± 0.06^C^	0.49 ± 0.05	0.41 ± 0.08
120ND	0.56 ± 0.07	0.55 ± 0.06	0.55 ± 0.10	0.70 ± 0.08^C^	0.71 ± 0.10	0.60 ± 0.13	0.36 ± 0.05	0.36 ± 0.04	0.31 ± 0.05	0.48 ± 0.06^C^	0.47 ± 0.05^C^	0.39 ± 0.06
180D	0.58 ± 0.09	0.54 ± 0.06	0.50 ± 0.08	0.78 ± 0.10^C^	0.73 ± 0.09^C^	0.60 ± 0.08	0.35 ± 0.05	0.34 ± 0.04	0.33 ± 0.06	0.49 ± 0.05^C^	0.47 ± 0.04^C^	0.40 ± 0.06
180ND	0.52 ± 0.07	0.51 ± 0.06	0.52 ± 0.10	0.71 ± 0.08^C^	0.69 ± 0.10	0.60 ± 0.10	0.33 ± 0.04	0.32 ± 0.04	0.33 ± 0.06	0.47 ± 0.05^C^	0.45 ± 0.04	0.40 ± 0.06

*C denotes difference from group C at p < 0.05.*

### Bilateral Ratios

#### Boxers Versus Control Group

**FIGURE 3:**
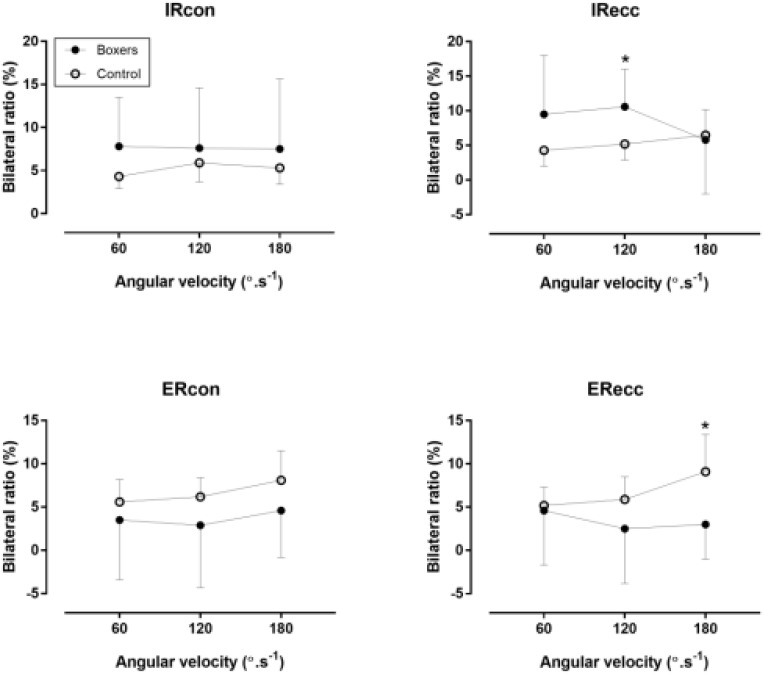
Bilateral ratio of internal (IR) and external rotators (ER) of shoulder against 60, 120, and 180°/s in boxers and control group. ^∗^difference between boxers and control group at *p* < 0.05.

Boxers had higher BL than control group (*p* < 0.05) in IRcon at 180°/s (η^2^ = 0.10) and IRecc at 120°/s (η^2^ = 0.16), whereas no difference was found in the other speeds (*p* > 0.05, η^2^ ≤ 0.07) (Figure 3). On the contrary, boxers had lower BL than control group in ERcon at 180°/s and no difference was observed in the speeds of ERcon and ERecc.

#### Boxers by Performance Level

With regards to performance groups, elite boxers and B scored higher than C (*p* < 0.05, η^2^ ≥ 0.16) at the high speed of IRcon, IRecc and ERecc (Table 5), whereas no difference was shown in the other speeds. Considering the comparison between limbs, peak torque in most conditions (60, 120, and 180 angular velocity; internal and external rotation; concentric and eccentric) was higher in D than in ND for Elite (except in ER60con and ER120ecc) and B performance group (except in ER120ecc), whereas D and ND differed only in IR60ecc for group C (Table 3).

**Table 5:** Bilateral ratios (%) in internal and external rotators of shoulders by performance group.

IR						ER					
Con			ecc			con			ecc		
Elite	B	C	Elite	B	C	Elite	B	C	Elite	B	C
8.6 ± 6.1	8.1 ± 5.5	5.1 ± 4.5	3.3 ± 7.5	3.6 ± 5.4	4.2 ± 7.9	10.1 ± 8.6	10.7 ± 9.7	6.0 ± 6.2	4.1 ± 5.6	5.7 ± 6.2	4.4 ± 9.2
9.3 ± 6.9	7.3 ± 7.1	2.4 ± 5.2	2.9 ± 7.4	4.8 ± 6.1	-0.2 ± 7.9	12.3 ± 4.6	9.7 ± 5.4	6.7 ± 6.2	1.8 ± 5.5	3.4 ± 6.6	3.2 ± 9.0
11.3 ± 6.3^∗^	6.3 ± 7.0^∗^	-2.8 ± 5.2	5.5 ± 4.9^∗^	5.9 ± 5.8	-0.1 ± 4.6	7.9 ± 7.8	6.0 ± 5.2	-0.8 ± 8.6	3.7 ± 3.7^∗^	3.9 ± 3.4^∗^	-1.0 ± 3.6

*^∗^difference from C at p < 0.05. IR, internal rotation; ER, external; con, concentric; ecc, eccentric.*

### Unilateral Ratios

#### Boxers Versus Control Group

**FIGURE 4:**
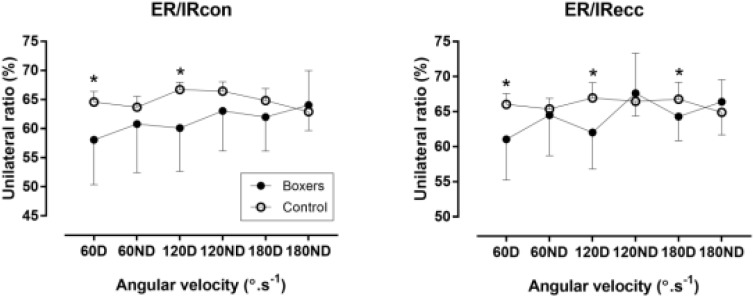
Unilateral ratio of internal (IR) and external rotators (ER) of shoulder against 60, 120, and 180°/s in boxers and control group. ^∗^difference between boxers and control group at *p* < 0.05; D, dominant limb; ND, non-dominant limb.

Unilateral was lower in boxers than in control group in ER/IRcon at 60D and 120D, and in ER/IRecc at 60D, 120D and 180D (*p* < 0.05, η^2^ ≥ 0.13) (Figure 4).

#### Boxers by Performance Level

No difference was observed in UL among performance groups (*p* > 0.05, η^2^ ≤ 0.08) (Table 6).

**Table 6:** Unilateral ratios of internal (IR) and external rotators (ER) of shoulder against 60, 120, and 180°/s among performance groups (Elite, B and C) of boxers.

	ER/IRcon			ER/IRecc		
	Elite	B	C	Elite	B	C
60D	57.2 ± 8.0	60.4 ± 8.1	57.3 ± 6.5	61.6 ± 5.4	60.5 ± 6.9	60.3 ± 6.1
60ND	60.5 ± 8.8	63.2 ± 7.1	57.9 ± 8.7	65.8 ± 5.3	63.9 ± 5.3	61.3 ± 7.4
120D	59.6 ± 7.5	63.4 ± 7.2	56.5 ± 6.3	61.2 ± 5.1	62.3 ± 5.7	64.1 ± 4.8
120ND	63.6 ± 6.3	65.1 ± 6.5	58.0 ± 7.7	68.5 ± 5.4	66.5 ± 5.3	66.5 ± 7.5
180D	60.9 ± 5.6	62.5 ± 6.6	64.7 ± 5.4	63.8 ± 3.2	63.7 ± 3.8	66.7 ± 2.7
180ND	64.9 ± 5.1	63.0 ± 7.7	63.0 ± 5.5	66.8 ± 2.9	65.1 ± 4.0	67.0 ± 2.1

*D, dominant limb; ND, non-dominant limb.*

### Functional Ratios

#### Boxers Versus Control Group

**FIGURE 5:**
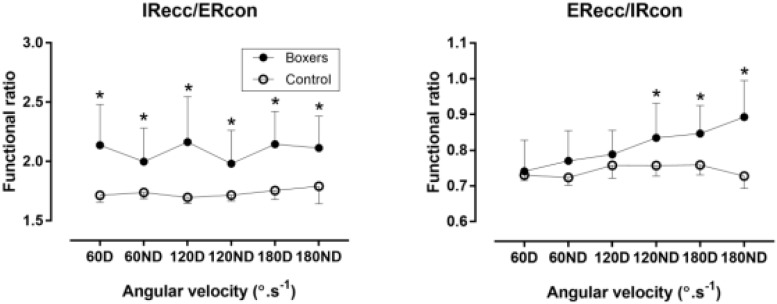
Functional ratio against 60, 120, and 180°/s in boxers and control group. ^∗^difference between boxers and control group at *p* < 0.05; D, dominant limb; ND, non-dominant limb.

A main effect of sport on IRecc/ERcon was observed with boxers presenting higher functional ratio than control group (*p* < 0.05) at 60D (η^2^ = 0.23), 60ND (η^2^ = 0.14), 120D (η^2^ = 0.23), 120ND (η^2^ = 0.16), 180D (η^2^ = 0.3) and 180ND (η^2^ = 0.22) (Figure 5). The difference between boxers and control group was larger for the D compared to ND. In addition, a main effect of sport on ERecc/IRcon was shown with boxers outscoring control group in the high speeds (*p* < 0.05).

#### Boxers by Performance Level

With regards to performance group, Elite and B had higher functional ratio than C for 180 of IRecc/ERcon, and 60ND, 120ND, and 180ND of ERecc/IRcon (*p* < 0.05, η^2^ ≥ 0.17) (Table 7).

**Table 7:** Functional ratios against 60, 120, and 180°/s by performance group (Elite, B and C).

	IRecc/ERcon			ERecc/IRcon		
	Elite	B	C	Elite	B	C
60D	2.14 ± 0.25	2.22 ± 0.54	1.99 ± 0.19	0.74 ± 0.09	0.76 ± 0.08	0.70 ± 0.09
60ND	1.99 ± 0.28	2.03 ± 0.34	1.96 ± 0.24	0.78 ± 0.07^∗^	0.81 ± 0.08^∗^	0.69 ± 0.09
120D	2.22 ± 0.37	2.12 ± 0.48	2.04 ± 0.25	0.79 ± 0.07	0.81 ± 0.06^∗^	0.73 ± 0.04
120ND	1.99 ± 0.24	2.00 ± 0.35	1.92 ± 0.31	0.86 ± 0.09^∗^	0.85 ± 0.07^∗^	0.73 ± 0.07
180D	2.21 ± 0.25^∗^	2.19 ± 0.25^∗^	1.87 ± 0.25	0.85 ± 0.07	0.86 ± 0.07	0.80 ± 0.11
180ND	2.15 ± 0.22^∗^	2.19 ± 0.33^∗^	1.87 ± 0.18	0.93 ± 0.08^∗^	0.89 ± 0.09	0.79 ± 0.12

*^∗^difference from C at p < 0.05. D, dominant limb; ND, non-dominant limb.*

## Discussion

### Peak Torque

The PT of boxers in IR and ER were stronger than the control group that affects the importance of these muscles on the strength of the boxers and on the performance of the athletes with similar kinetic chain (Fleisig et al., 1999). When comparing the PT in the athletes groups the elite level presented higher values in both contractions and agree with previous research (Filimonov et al., 1985), where the masters and candidates of masters of sports generate more maximum and powerful punches than the other level groups. The higher PT in the elite group might be due to an optimal synchronization of the kinetic chain (leg, trunk, arm) and the neuromuscular stimulus of training (Verkhoshansky and Verkhoshansky, 2011). It has been observed previously that beginner athletes developed power 15% at 50 ms and their advanced peers 28% in this time (Tillin et al., 2010), whereas the more experienced athletes produced higher values of strength (Chandler et al., 1992).

The PT were higher in the dominant limb with a statistical significant difference at the three speeds, stressing the importance of this edge in coaching and training performance (Tasiopoulos et al., 2015), because the rear hand (normally is the strongest limb) produce more powerful punches while the lead arm hits more times (Slimani et al., 2017) and dependent on the tactics of the games while the athletes produces more energy on the second and third round (Hanon et al., 2015).

### Bilateral Ratios

The bilateral ratios were approximately 6–10% for the IR and about 3–6% for the ER which are below 10% and are considered normal values for the myodynamic ratios (Dvir, 2004) with low risk of injuries, except the IR ECC on 120°/s thus as a medium and maybe nonfunctional speed. Also, it is in agreement with the sports of symmetrical movements (Batalha et al., 2012) and highlighted the importance of the rotators muscles on training.

### Unilateral Ratios

The UL ratios of CON and ECC were lower (0.58–0.64) at all speeds except of 120 and 180°/s ND ECC, which considered below normal of 0.66 of concentric with injury risk (Wathen, 1994), with the range of overhead athletes between 0.46 and 1.05 (Berckmans et al., 2017), but with a trend to increase at higher speeds, stressing the differences of the strength of the dominant limb of the IR at low speed, and the greater power of ER at high speed.

### Functional Ratios

The functional ratios of IR ECC/ER CON of the men athletes (2.0–2.2) were statistical significant with the control group ND at all speeds, and highlights the importance of both limbs in this phase of kinetic chain especially at the high speed because both limbs were with no statistical significant similar ratios. Also, the ratios of men athletes of ER ECC/IR CON (0.74–0.89) were statistical significant with the control group at 180°/s and between D and ND at all speeds, which maybe link with the comeback of the limb.

The low bilateral ratios of the IR contractions explain the support of the rotators on the performance which is the contribution of the fast movements of the punches on the target specially of the dominant limb with higher strength and the very low ratios of ER explain the possibility of both muscles to produce more power and faster returns of the punches. In the initial phase of the punch the movement action became from the contractions of the functional ratio of IR ECC/ER CON which is the acceleration and the next phase with the deceleration ratio of ER ECC/IR CON which is the comeback of the punch. Thus, the importance of the training of rotator muscles was highlighted using an optimal program of shoulder exercises for the evaluation, prevention, and rehabilitation and strength conditioning (Ratamess, 2012).

A limitation of the study was that it was conducted in the preparation period of the training year. Muscle strength increases during preparation (Bompa and Buzzichelli, 2015); thus, it would be reasonable to assume that differences between boxers and non-athletes would vary during the training year. On the other hand, strength of the present research was its novelty as it was the first to examine PT, BL, UL, and functional ratios of rotator muscles of the shoulder in boxers by performance level and non-athletes. Considering the popularity of boxing (Chaabène et al., 2014), our findings would be of great practical interest for coaches and fitness trainers in the context of training monitoring.

## Conclusion

Boxers in the pre-season had normal myodynamic BL ratios on the rotators of shoulders but were at injury risk at the UL ratios. This observation highlighted the importance of these muscles for training and performance, with the reduction of deficit arises of the neuromuscular adaptations of strength training and the importance of the evaluation of asymmetries in athletes as according to the motor each sport templates which is an adaptive necessity for better performance and the prevention of injury. Further studies in higher speeds 240, 300°/s and in women athletes are necessary to confirm the contribution of the rotators muscles to performance and to the healthy exercise.

## Author Contributions

IT performed the laboratory analyses, the statistical analyses, and drafted the manuscript. PN, AT, AS, TR, and BK helped in drafting the manuscript.

## Conflict of Interest Statement

The authors declare that the research was conducted in the absence of any commercial or financial relationships that could be construed as a potential conflict of interest.
